# The effects of maternal preeclampsia on inflammatory cytokines and clinical outcomes in premature infants

**DOI:** 10.12669/pjms.36.2.1316

**Published:** 2020

**Authors:** Salih Cagri Cakir, Bayram Ali Dorum, Nilgun Koksal, Hilal Ozkan

**Affiliations:** 1 Salih Cagri Cakir, Medical Doctor, Department of Pediatrics, Division of Neonatology, Uludag University Medical Faculty, Gorukle, 16059, Nilufer–Bursa–Turkey; 2 Bayram Ali Dorum, Department of Pediatrics, Division of Neonatology, Uludag University Medical Faculty, Gorukle, 16059, Nilufer–Bursa–Turkey; 3 Prof. Nilgun Koksal, Medical Doctor, Department of Pediatrics, Division of Neonatology, Uludag University Medical Faculty, Gorukle, 16059, Nilufer–Bursa–Turkey; 4 Hilal Ozkan, Associate Professor, Department of Pediatrics, Division of Neonatology, Uludag University Medical Faculty, Gorukle, 16059, Nilufer–Bursa–Turkey

**Keywords:** Preeclampsia, Premature, Newborn, Cytokine, Cord blood

## Abstract

**Objective:**

To investigate the effects of maternal preeclampsia on inflammatory cytokines and neonatal outcomes in premature infants.

**Methods:**

The study included preterm infants born at gestational age ≤32 weeks in a tertiary university hospital between January 2016 and January 2017. The study group consisted of infants born from mothers with preeclampsia (Group-1), and the control group consisted of infants born from normotensive mothers (Group-2). Demographic characteristics and clinical outcomes of the infants were recorded. IL-6, IL-8, IL-10, and TNF-α cytokine levels were measured from umbilical cord blood samples.

**Results:**

A total of 108 infants were included in the study, of which 34 were in the Group-1 and 74 in the Group-2. Gestational ages (29 vs 30 weeks) of the infants in both groups were similar. There was no significant difference between the cytokine levels of infants with and without preeclampsia. The rate of small for gestational age, retinopathy of prematurity, intraventricular hemorrhage, necrotizing enterocolitis, neutropenia, and thrombocytopenia were significantly higher at the infants with preeclampsia.

**Conclusion:**

Maternal preeclampsia leads to an increase at the neonatal morbidities in premature infants without causing a significant alteration at the cytokine levels in cord blood.

## INTRODUCTION

Preeclampsia, which may lead to maternal and perinatal morbidity and mortality, affects 1-7% of pregnancies.^1^ Preeclampsia is a complication characterized by hypertension associated with proteinuria or other end-organ damage after 20 weeks of gestation.^2^ There is a complex physiopathology in preeclampsia that affects many organs as a result of inflammatory processes progressing through cytokines and endothelial cell activation.^2^ Preeclampsia causes placental insufficiency and uteroplacental incompatibility.^2^ As a result of fetoplacental effects, intrauterine growth restriction, oligohydramnios, loss of end diastolic flow, and preterm delivery can occur.^2^ The clinical condition of the mother due to preeclampsia is uncertain in predicting fetal or neonatal outcomes.^2^

Cytokines are the general name of small protein structure molecules involved in immune, inflammation, and hematopoiesis and are divided into two as proinflammatory and anti-inflammatory effects.^3^ Interleukin (IL) 6, IL-8 and tumor necrosis factor α (TNF-α) are proinflammatory cytokines, and IL-10 is an anti-inflammatory cytokine.^3^ The balance between cytokines affects fetal and neonatal outcomes.^3^

Different results have been reported on inflammatory cytokines which play an essential role in preeclampsia.^4^ Also, studies report that inflammatory mediators and proinflammatory cytokines increase in premature births.^3^ Changes in cytokines are associated with preterm delivery and poor neonatal outcomes.^3^ The cytokine balance in premature infants who born from mothers with preeclampsia should be examined regardless of the effect of prematurity.^3^

We have hypothesized that preeclampsia may alter the balance between anti-inflammatory and proinflammatory cytokine levels in premature babies. In this prospective study, we aimed to investigate the effects of maternal preeclampsia on inflammatory cytokines and neonatal outcomes in premature infants.

## METHODS

**Fig.1:**
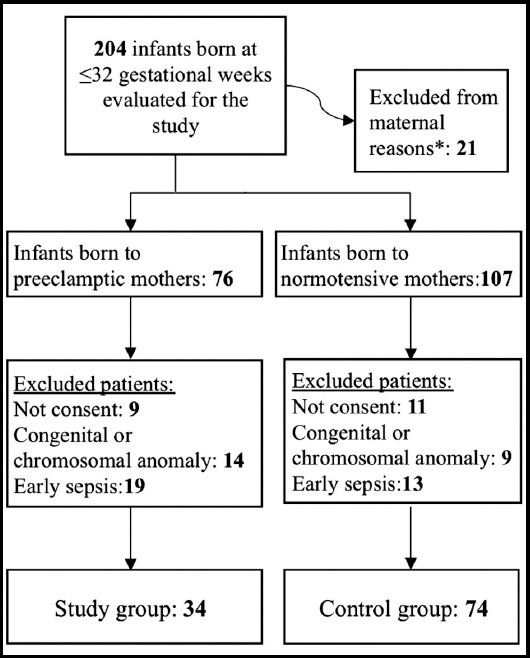
Flowchart of the participation of patients.

This study was conducted at a tertiary university hospital between January 2016 and January 2017. Premature infants born at a gestational age of ≤ 32 weeks were included in the study. Written and verbal consent was obtained from the parents. Infants who were born to mothers with diabetes mellitus, chorioamnionitis, and early membrane rupture were excluded from the study. Babies with early sepsis (who had high acute phase reactants in the first 72 hours or positive blood culture) and who had chromosomal or major congenital anomalies were excluded from the study. A flowchart with the participation of patients in the study is shown in Fig.1.

The study was approved by the ethics committee (Ref. No: 2014-2 / 24 dated January 21, 2014) of Uludag University Medical Faculty.Clinical features including gender, birth weight, gestational age, type of delivery, neonatal morbidities, antenatal steroid application and preeclampsia were recorded. The study group consisted of babies with preeclampsia and the control group consisted of babies with normotensive mothers. After 20 weeks of gestation, ≥140 / 90 mm Hg blood pressure with 300 mg/day proteinuria was defined as preeclampsia.^2^

Respiratory distress syndrome (RDS)^5^, intraventricular hemorrhage (IVH)^6^, bronchopulmonary dysplasia (BPD)^7^, necrotizing enterocolitis (NEC)^8^, prematurity retinopathy (ROP)^9^ and patent ductus arteriosus (PDA)^10^ diagnoses were made according to the criteria defined in the literature.

Gestational ages were evaluated according to modified Ballard scoring.^11^ The birth weights of babies were evaluated according to Fenton preterm growth chart, and infants who have weights <10 percentile were defined as small for gestational age infants (SGA).^12^

### Collection and analysis of blood samples

Umbilical cord blood samples were taken immediately after delivery. These samples were kept for 30 minutes and then centrifuged at 2000 x g for ten minutes. Serum samples were stored at -20 °C until measurement. IL-6, IL-8, IL-10, and TNF-α cytokine levels were measured by enzyme-linked immunosorbent assay (ELISA).

The leukopenia and thrombocytopenia were evaluated by leukocyte and platelet counts on the 1st-day complete blood count of the patients. Thrombocytopenia was defined as a platelet count of <150.000/mm.^3^,^13^ Leukopenia and neutropenia situation were evaluated according to Manroe and Rodwell scoring system.^14^,^15^ Leukopenia was defined as leukocyte count was <5000/mm^3^.

### Statistical Analysis

The findings of the study were analyzed using the SPSS version 23 program with an appropriate statistical method. Chi-square test was used for categorical variables. Shapiro-Wilk test was used to compare the distribution of two independent samples in groups, while those with normal distribution were compared with the t-test and those without a normal distribution were compared with the Mann Whitney U test. P values <0.05 were considered to be statistically significant.

## RESULTS

During the study period, 108 infants who met the study criteria of 204 premature infants born at ≤32 weeks of gestational age were included in the study (Fig.1). Demographic characteristics of the patients are similar (Table-I). The clinical features, neonatal morbidity and mortality rates of the groups are summarized in Table-II. The rates of ROP (41%, 16%, p = 0.003) and SGA (23% to 8%, p = 0.027) in the infants of preeclamptic mothers were found to be significantly higher than the other premature babies.

**Table-I:** Demographic characteristics of the study and control groups.

Demographic features	Study group, n= 34	Control group, n= 74	P value
Gestational age (week), median (min-max)	29 (26-32)	30 (27-32)	0.1^&^
Birth weight (gr), median (min-max)	995 (585-1820)	1290 (630-2200)	**<0.001^&^**
Male gender, n (%)	14 (41)	36 (49)	0.4*
Caesarean section n (%)	30 (88)	68 (92)	0.5*
Antenatal steroid, n (%)	26 (76)	58 (78)	0.8*
Apgar 1st minute, median (min-max)	4 (1-7)	6 (1-9)	**<0.001^&^**
Apgar 5th minute, median (min-max)	7 (3-9)	8 (4-9)	**<0.001^&^**

* Chi-square test was used,

& Mann Whitney U test was used.

**Table-II:** Clinical features of the study and control groups.

Clinical features	Study group n= 34	Control group n= 74	P value
Respiratory distress syndrome, n (%)	26 (76)	52 (70)	0.5*
Patent ductus arteriosus, n (%)	16 (47)	36 (49)	0.6*
Small for gestational age, n (%)	8 (23)	6 (8)	0.027*
Bronchopulmonary dysplasia, n (%)	8 (23)	22 (30)	0.5*
Mild 1, n (%)	4 (12)	12 (16)	NA
Moderate 2, n (%)	2 (6)	4 (5)	NA
Severe 3, n (%)	2 (6)	6 (8)	NA
Retinopathy of prematurity, n (%)	14 (41)	12 (16)	0.003*
Stage 1, n (%)	4 (12)	6 (8)	NA
Stage 2, n (%)	4 (12)	2 (3)	NA
Stage 3, n (%)	6 (18)	4 (5)	NA
Intraventricular hemorrhage, n (%)	14 (41)	20 (27)	0.1*
Grade 1, n (%)	10 (29)	14 (19)	NA
Grade 2, n (%)	3 (9)	4 (5)	NA
Grade 3, n (%)	1 (3)	2 (3)	NA
Grade 4, n (%)	0 (0)	0 (0)	NA
Necrotizing enterocolitis, n (%)	6 (18)	6 (8)	0.2*
Stage 2, n (%)	4 (12)	2 (3)	NA
Stage 3, n (%)	2 (6)	4 (5)	NA
Day of hospitalization, median (min-max)	49 (14-104)	33 (14-167)	**0.019^&^**
Mortality rate, n (%)	2 (6)	8 (11)	0.4*

* Chi-square test was used,

& Mann Whitney U test was used.

In the study and control groups, umbilical cord blood cytokine levels and leukocyte, neutrophil and platelet counts on day one are given in Table-III. There were no significant differences in cytokine levels between preeclamptic infants and other premature infants. While there was no difference between the groups regarding leukocyte counts and leukopenic patient numbers, the rates of neutropenia (18%, 5%, p = 0.042) and thrombocytopenia (47% to 13%, p <0.0001) in the preeclamptic group were significantly higher than the other premature babies.

**Table-III:** Umbilical cord cytokine levels, and first day leukocyte, neutrophil and platelet counts of infants.

	Study group n= 34	Control group n= 74	P value
IL-6, median (min-max) pg/ml	17 (0-267)	23 (0-243)	0.2^&^
IL-8, median (min-max) pg/ml	174 (10-1020)	159 (10-989)	0.3^&^
IL-10, median (min-max) pg/ml	6 (0-224)	3 (0-202)	0.2^&^
TNF-alfa, median (min-max) pg/ml	5.7 (2.5-23.7)	5.3 (1.3-26.6)	0.4^&^
Leukocytes count, median (min-max) /mm^3^	10400 (2230-23200)	8590 (3660-42700)	0.6^&^
Neutrophil count, median (min-max) /mm^3^	2559 (669-5940)	3300 (1000-8190)	**0.015**^&^
Platelet count, median (min-max) /mm^3^	152000 (22900-424000)	184000 (64400-388000)	**0.003**^&^
Patients with leukopenia, n (%)	6 (18)	6 (8)	0.1*
Patients with neutropenia, n (%)	6 (18)	4 (5)	0.042*
Patients with thrombocytopenia, n (%)	16 (47)	10 (13)	<0.0001*

* Chi-square test was used,

& Mann Whitney U test was used.

## DISCUSSION

Preeclampsia continues to be one of the important causes of perinatal mortality and morbidity.^16^ Maternal preeclampsia is reported to be associated with morbidity especially in premature infants.^16^ According to our knowledge, for the first time in the literature, the levels of IL-6, IL-8, IL-10 and TNF-α in the cord blood of premature infants born at ≤32 weeks of gestational age from mothers with preeclampsia and normotensive mothers were compared. No significant difference was found between the cytokine levels of babies in both groups. Although maternal preeclampsia affects cytokine levels, the difference may not be detected because cytokine levels are changed by other conditions causing premature delivery.

Different results have been reported in studies that investigate the clinical effects of maternal preeclampsia on premature infants. In the literature, there are different studies reporting that birth weight and Apgar scores are lower in babies of preeclamptic mothers while thrombocytopenia, neutropenia, SGA, ROP, BPD and NEK ratios are higher.^13^,^16^-^19^ It is also reported that the babies of preeclamptic mothers stay longer in the hospital.^17^ In our study, Apgar scores and birth weights of the babies of preeclamptic mothers were found to be significantly lower. Also, ROP and SGA rates were significantly higher in the babies of preeclamptic mothers. Besides, we found that the babies of preeclamptic mothers were hospitalized for longer. Also, the rates of IVH (41% vs. 27%) and NEC (18% vs. 8%) in the infants of preeclamptic mothers were found higher, although not statistically significant. All these results are similar to the adverse clinical effects reported in the literature.

Preeclampsia can have different effects on more than one system.^3^ The pathophysiology of these effects is not fully known.^3^ It has been emphasized that preeclampsia may cause these effects by causing a change in inflammatory and anti-inflammatory cytokine levels.^3^ The balance between proinflammatory cytokines (IL-6, IL-8, TNF-α) and anti-inflammatory cytokines (IL-10) affects neonatal morbidities since the antenatal period.^20^

Also, there is a positive correlation between IL-6, IL-8, and TNF-α.^21^ Different results have been reported in the literature regarding the levels of IL-6, IL-8, and TNF-α in the cord of infants of preeclamptic mothers. Tosun et al. reported that higher IL-6, IL-8, and TNF-α levels in infants of preeclamptic mothers than normotensive mothers’.^22^ Guillemette et al.^4^ reported that the increase of TNF-α levels in the infants of preeclamptic mothers while Kupferminc et al.^23^ reported that the decrease of TNF-α levels. Catarino et al. showed that there was no difference in IL-6 and TNF-α levels.^24^ These studies have included term and preterm infants and also infants had an infection. In our study, only infants ≤ 32 weeks of gestational age and without infection were enrolled. In our study, no difference was found between cord blood IL-6, IL-8, and TNF-α levels of the babies of preeclamptic mothers and normotensive mothers.

IL-10 plays a crucial role in the physiopathology of preeclampsia as an immunomodulator. Low levels of IL-10 were associated with preeclampsia.^25^ Also, the rate of TNF-α / IL-10 has been reported to be increased in placenta samples of preeclamptic mothers.^26^ There is no study stating the cord blood IL-10 level in premature infants of preeclamptic mothers. In our study, no significant difference was found between IL-10 levels in the cord blood of the babies of preeclamptic mothers and normotensive mothers. In preeclampsia, inflammatory cytokines are released with a decrease in uteroplacental blood flow and hypoxia.^16^ These cytokines and endothelial cell dysfunction cause a systemic inflammatory response.^16^ All these processes can cause harmful effects on fetal and neonatal outcomes.^16^ Although the cause of preeclampsia is not fully understood, the immune and vascular events play a role at the pathogenesis.^16^ Here, IL-10 is effective in controlling inflammation and regulating vascular function.^25^ The impaired biological mechanisms of angiogenesis in the intrauterine period at preeclampsia and its effects on preterm infants are not known exactly.^16^

### Limitation of the study

It includes a limited number of cases. Another limitation of this study is that we measured a small number of cytokine levels as a marker of inflammation.

## CONCLUSION

As a result of maternal preeclampsia, birth weight and APGAR scores of preterm infants were significantly lower, and length of hospital stay, SGA and ROP rates were higher. Besides, the rates of IVH (41% vs. 27%) and NEC (18% vs. 8%) were higher in the infants of preeclamptic mothers, although not statistically significant. However, levels of TNF alpha, IL-6, IL-8 and IL-10 in premature infants were found to be unaffected by the mother’s preeclampsia condition. In this study, it was shown that maternal preeclampsia leads to an increase at the neonatal morbidities in premature infants without causing a significant alteration at the cytokine levels in cord blood. Studies are needed to explain the physiopathological basis of the adverse clinical effects of preeclampsia in premature infants.

### Authors’ Contribution:

**SCC, BAD, HO, NK:** Conceived, designed and did statistical analysis & editing of manuscript, are responsible for integrity of research.

**SCC, BAD:** Did data collection and manuscript writing.

**BAD, SCC, HO, NK:** Did review and final approval of manuscript.
